# Supplementary material for: Characterization of PARP6 Function in Knockout Mice and Patients with Developmental Delay
Source: Cells. 2021 May 22;10(6):1289. doi: 10.3390/cells10061289 (PMC8224619; doi:10.3390/cells10061289)
Supplement: Supplementary file 1 [file cells-10-01289-s001.zip › cells-1196478-supplementary.pdf]

Table S1: List of PCR and sequencing primers, as well as shRNA oligos used

Gene	Forward	Reverse	Size (bp)
Parp6 (CRISPR seq)	gctacacaaagaaaccctgtct		
Parp6$^{+/+}$WT	ggggaagagacagcacagat	ggggaagagacagcacagat	743
Parp6$^{-/-}$indel	gcacagtggccattgactag	aaagagctggaattggggct	1495
SRY-Male	ttgtctagagagcatggagggccatgtcaa	ccactcctctgtgacactttagccctccga	273
Cacna2d1-PCR	ccagagcaaactcccaggtc	ccagagcaaactcccaggtc	534
Cacna2d1-SEQ	gggcatcagaacccatagca		
Pstpip1-PCR	caccatatggggaagctgca	ctggcctgcctatatgctcc	502
Pstpip1-SEQ	aaacaggcctccttgtggtc		
Rims2-PCR	gtcccctgagccattggtac	gcagatgccacacactttgg	502
Rims2-SEQ	aagagcccatgttggagagc		
Atp2b2-PCR	ggcaggagtcaagcaggaat	tgcctcctgatcggtgacta	529
Atp2b2-SEQ	ggtaccattctccaggcagg		
Zfp708-PCR	tgccagagttgctgtaccaa	gcacaaactgatccaagtggg	587
Zfp708-SEQ	agaagcctacacgataggagag		
Parp6 shRNA	atgacccagggctcatatTCTCTTGAAata tgagccctgggtcatc		
LacZ shRNA	actaccgttgttataggtgtc		
RT-PCR Parp6 set 1	gagaactccgtgtccatcag	accgtagcctcaacacaatag	142
RT-PCR Parp6 set 2	tccacctttactttcacctacg	caggaattgggtctgtagtgac	142
RT-PCR HPRT	ccccaaaatggttaaggttgc	tgaagtactcattatagtcaagggc	137
Parp6 pCMV	taggccaccggtaccatggtgagcaa	tacttatctagattagtttgtgtaaacctgagttccg a	2532
Parp6 extra neuronal	agctagggtaccatcaagaagttccgagc	ctagctggatccaacttgtccatctgtttc	665
Parp6 point Mut	agatgcggatcccttggcccatcct	caatttacgcgttaagatacattgatgagtttggac aaaccac	915
Parp6 trunc	agatgcggatcccttggcccatcct	caatttacgcgttcagtttgtgtaaacctga	568

Table S2: List of antibodies used. All primary antibodies were diluted to working concentrations in 3% bovine serum albumin in TBST 100mM TBS + 0.1% Tween-20) for immunoblot or 10% bovine serum albumin in PBST (100mM PBS + 1% Triton-X100) for immunofluorescence. All secondary antibodies were diluted to working concentrations in 5% milk-TBST for immunoblot or 10%-PBST for immunofluorescence.

Antibody	Company	Catalogue Nr	species	dilution	
anti-PARP6	Sigma Prestige®	HPA026991	Rb pAb	1:1000	1˚
anti-Parp6	gift from Paul Chang		Rb pAb	1:1000	1˚
anti-β-actin (C4)	Santa Cruz Biotechnology	sc-47778	Ms mAb	1:1000	1˚
anti- β –tubulin III	Sigma-Aldrich	T2200	Rb pAb	1:1000	1˚
anti-GFP	Chromotek	PABG1-100	Rb pAb	1:2000	1˚
Anti-neurofilament (2H3)	DSHB	RRID: AB-531793	Ms mAb	1:500	1˚
HRP-conjugated streptavidin	Jackson ImmunoResearch	016-030-084	reagent	0.4 μg/mL	1˚
anti-mouse IgG, HRP-conjugated	Thermo Fisher Scientific	62-6520	goat	1:5000	2˚
anti-rabbit IgG, HRP-conjugated	Cell Signaling Technology	7074s	goat	1:1000	2˚
anti-mouse IgG Alexa Fluor 488-conjugated	Thermo Fisher Scientific	A-21202	donkey	1:1000	2˚

Table S3: **Parp6 nucleotide sequences** of extra neuronal insert, C563R and R485H point mutations, and the truncated version of Parp6 designed for Parp6 cloning.

Long neuronal sequence	aagaggcacagttggttcaaggcaagtggtaccatcaagaagttccgagct
Parp6^{R485H}	cgc485cac
Parp6^{C563R}	tgt563aga
sgRNA	ggcattcaatcctgcgcaatGGG
CRISPR mutation	cattcaatcc(-tgcgc,+a)aat → cattcaatccaaat
Parp6^{TR}	gcattcaatccaaatgggctggtcaatgcatcctacaccaaactgcagctgcatggagcagcctatggcaaaggcatctacc**tga**(*STOP*)

atggacatcaaaggccagttctggaatgatgatgattcagaaggagataatgaatcagaggaatttctgtatggagttcaggggagctgtgcagctgacct
gtatagacacccacagcttgatgcagacattgaagcagtgaaagagatctacagtgagaactccgtgtccatcagagaatatggaactatcgatgacgtgg
accttgacctccacatcaacatcagcttcctcgatgaggaagtatctacagcttggaaggtccttcgaacagaacctattgtgttgaggctacggttttctctctct
cagtaccttgatggaccagagccatcaattgaggttttccagccatcgaataaggaagggtttgggctaggtcttcagttgaaaaaaatcctgtgtatgtttac
atcccaacaatggaagcatctgagcaatgatttcttgaagacccagcaggagaagaggcacagttggttcaaggcaagtggtaccatcaagaagttccga
gctggcctcagcatcttctcacccattcccaagtctcccagttttcctatcatacaggactctatgctgaaaggcaaactgggtgtaccagagctccgagttgga
cgccttatgaatcgttccatttcttgcaccatgaagaacccaaaagtagaggtgtttggctatcccccagcccccaggcaggtctcctgtgcccccagcacgt
gggcctccctcccccagcacggacttctcctttggtcagtggtcactgcaagaacatccccactctggagtatggattccttgtccagattatgaagtatgcaga
gcagaggattccaacgttaaatgagtactgtgtggtttgtgatgagcagcatgtcttccagaatgggtccatgctcaagccagctgtctgtactcgtgagttat
gcgtcttctccttctacacactgggagtcatgtctggagctgcagaagaagtggctacaggagcagaggtggtagatctgctggtggccatgtgtagagca
gctttggaatcccctagaaaaagcatcatcttcgagccttatccctctgtggtggaccccactgatcccaagactctggcctttaaccctaagaagaagaattat
gagcggcttcagaaagctctggatagtgtgatgtccatccgggagatgacccagggctcatatttggaaatcaagaaacagatggacaagctggatccctt
ggcccatcctcttctgcagtggataatttctagcaacaggtcacacattgtcaaactacctctcagcaggctgaagttcatgcacacctcacaccaattcctcctg
ctgagcagccctcctgccaaggaggctcggttccggacagccaagaagctctacggaagcacctttgctttccatgggtcccacattgagaattggcattcaa
tcctgcgcaat**ggg**ctggtcaatgcatcctacaccaaactgcagctgcatggagcagcctatggcaaaggcatctacctgagccccatctccagtatttccttt
ggatactcaggaatgggcaaaggacagcacaggatgccttccaaggatgagctggtccagagatacaacaggatgaacaccatcccccagacccgatcc
attcagtcaaggttcctgcagagtcggaatctaaac**tgt**atagcactttgtgaagtgattacgtctaaggacctccagaagcatgggaacatttgggtgtgccc
tgtgtctgaccatgtctgcacaaggttcttctttgtatatgaggatgggcaggtgggcgatgccaacattaatactcaggaccccaagattcagaaggaaatc
atgcgtgtgatcggaactcaggtttacacaaactga

Table S4: Parp6 Amino acid sequence of Parp6^{WT} (Wild Type, top) and Parp6^{TR} (CRISPR mutant, bottom) showing extra neuronal sequence (red), catalytic domain (blue), localization of clinical P1211L, H256Y, R485H/C and C563R point mutations (yellow highlight), CRISPR indel (☼), frameshift mutation (gray highlight) and early termination codon (*).

**Parp6^{WT}**	MDIKGQFWNDDDSEGDNESEEFLYGVQGSCAADLYRHPQLDADIEAVKEIYS<br>ENSVSIREYGTIDDVDLDLHINISFLDEEVSTAWKVLRTEPIVLRLRFSLSQYLD<br>GPEPSIEVFQPSNKEGFGLGLQLKKILCMFTSQQWKHLSNDFLKTQQEKRHS<br>WFKASGTIKKFRAGLSIFSPIPKSPSFPIIQDSMLKGKLGVPELRVGRLMNRSISC<br>TMKNPKVEVFGYPPSPQAGLLCPQHVGLPPPARTSPLVSGHCKNIPTLEYGFL<br>VQIMKYAEQRIPTLNEYCVVCDEQHVFQNGSMLKPAVCTRELCVFSFYTLGV<br>MSGAAEEVATGAEVVDLLVAMCRAALESPRKSIIFEPYPSVVDPTDPKTLAFN<br>PKKKNYERLQKALDSVMSIREMTQGSYLEIKKQMDKLDPLAHPLLQWIISSNR<br>SHIVKLPLSRLKFMHTSHQFLLLSSPPAKEARFRTAKKLYGSTFAFHGSHIENW<br>HSI☼LRNGLVNASYTKLQLHGAAYGKGIYLSPISSISFGYSGMGKGQHRMPSK<br>DELVQRYNRMNTIPQTRSIQSRFLQSRNLNCIALCEVITSKDLQKHGNIWVCP<br>VSDHVCTRFFFVYEDGQVGDANINTQDPKIQKEIMRVIGTQVYTN
**Parp6^{TR}**	MDIKGQFWNDDDSEGDNESEEFLYGVQGSCAADLYRHPQLDADIEAVKEIYS<br>ENSVSIREYGTIDDVDLDLHINISFLDEEVSTAWKVLRTEPIVLRLRFSLSQYLD<br>GPEPSIEVFQPSNKEGFGLGLQLKKILCMFTSQQWKHLSNDFLKTQQEKRHS<br>WFKASGTIKKFRAGLSIFSPIPKSPSFPIIQDSMLKGKLGVPELRVGRLMNRSISC<br>TMKNPKVEVFGYPPSPQAGLLCPQHVGLPPPARTSPLVSG<u>**H**</u>CKNIPTLEYGFL<br>VQIMKYAEQRIPTLNEYCVVCDEQHVFQNGSMLKPAVCTRELCVFSFYTLGV<br>MSGAAEEVATGAEVVDLLVAMCRAALESPRKSIIFEPYPSVVDPTDPKTLAFN<br>PKKKNYERLQKALDSVMSIREMTQGSYLEIKKQMDKLDPLAHPLLQWIISSNR<br>SHIVKLPLSRLKFMHTSHQFLLLSSPPAKEARFRTAKKLYGSTFAFHGSHIENW<br>HSI☼QMGWSMHPTPNCSCMEQPMAKAST*

Table S5: Overall development and axonal outgrowth of embryonic stage E12.5 Parp6^{HT} and Parp6^{TR} was indistinguishable. Average measurements of eye and paw area (μm^2), paw outgrowth, abdominal innervation outgrowth and spinal cord thickness (between the 2nd and 3rd DRG) (μm). For explanation of measurements, please see methods.

Measurements	Parp6^{HT}		Parp6^{TR}		*p* value (2-tail t-test)
	Mean ± S.E.M.	N	Mean ± S.E.M.	N	
Eye Area (μm^2)	117818.13 ± 4188.22	8	103891 ± 8569.71	6	0.1876
Paw Area (μm^2)	450192.62 ± 67433.80	9	457936.27 ± 110986.52	3	0.9553
Paw Outgrowth (μm)	1480.92 ± 54.52	9	1344.02 ± 116.34	5	0.3276
Abdominal innervation outgrowth (μm)	1386 ± 62.62	9	1385.70 ± 94.68	5	0.9948
Spinal Cord Thickness (μm)	134 ± 19.40	9	101.14 ± 10.58	6	0.1541

Table S6: List of 89 putative proteins that interact with Parp6^{WT} in cortical neurons identified by LC-MS/MS. Table shows two sets of data (1 and 2), P6 (P): Myc-BirA*-Parp6^{WT}, GFP (G): Myc-BirA*-GFP.

DESCRIPTION	P6_1	GFP_1	P6_2	GFP_2	P(1+2)	G(1+2)	B-G
[BirA_PARP6]	29.00	0.00	28.00	0.00	57.00	0.00	57.00
Microtubule-associated protein 2	16.00	0.00	4.00	0.00	20.00	0.00	20.00
Microtubule-associated protein 1B	5.00	1.00	3.00	0.00	8.00	1.00	7.00
Microtubule-associated protein RP/EB family member 2	4.00	0.00	2.00	0.00	6.00	0.00	6.00
Microtubule-associated protein tau	3.00	0.00	3.00	0.00	6.00	0.00	6.00
Heat shock cognate 71 kDa protein	5.00	2.00	3.00	1.00	8.00	3.00	5.00
Ubiquitin-40S ribosomal protein S27a	2.00	1.00	2.00	1.00	4.00	2.00	2.00
Keratin, type II cytoskeletal 5	5.09	0.00	0.50	0.00	5.59	0.00	5.59
Tubulin alpha-1B chain	6.00	0.33	4.33	5.00	10.33	5.33	5.00
Actin, alpha cardiac muscle 1	0.00	0.00	3.20	0.00	3.20	0.00	3.20
60S ribosomal protein L3	2.00	0.00	1.00	0.00	3.00	0.00	3.00
Prelamin-A/C	2.00	0.00	1.00	0.00	3.00	0.00	3.00
Putative 60S ribosomal protein L37a	2.00	0.00	1.00	0.00	3.00	0.00	3.00
60S ribosomal protein L30	2.00	0.00	1.00	0.00	3.00	0.00	3.00
Annexin A2	2.00	0.00	1.00	0.00	3.00	0.00	3.00
Dihydropyrimidinase-related protein 2	3.00	1.00	1.33	1.00	4.33	2.00	2.33
60S ribosomal protein L32	3.00	1.00	2.00	2.00	5.00	3.00	2.00
Heat shock protein HSP 90-beta	2.00	1.00	1.00	0.00	3.00	1.00	2.00
Alpha-actinin-4	2.00	0.00	0.00	0.00	2.00	0.00	2.00
60S ribosomal protein L18	1.00	1.00	2.00	0.00	3.00	1.00	2.00
Elongation factor 1-alpha 2	3.50	0.00	0.00	2.00	3.50	2.00	1.50
60S ribosomal protein L27a	2.00	3.00	3.00	1.00	5.00	4.00	1.00
Cofilin-1	2.00	1.00	1.00	1.00	3.00	2.00	1.00
Ubiquitin carboxyl-terminal hydrolase isozyme L1	2.00	1.00	0.00	0.00	2.00	1.00	1.00
40S ribosomal protein S9	1.00	2.00	2.00	0.00	3.00	2.00	1.00
60S ribosomal protein L27	1.00	2.00	2.00	0.00	3.00	2.00	1.00
60S ribosomal protein L13	0.00	1.00	3.00	1.00	3.00	2.00	1.00
14-3-3 protein epsilon	0.00	1.00	2.00	0.00	2.00	1.00	1.00
40S ribosomal protein S7	3.00	4.00	2.00	1.00	5.00	5.00	0.00
Histone H2B type 1	2.00	3.00	2.00	1.00	4.00	4.00	0.00
Elongation factor 1-alpha 1	3.50	8.00	4.00	2.00	7.50	10.00	-2.50
Tubulin beta-5 chain	2.67	9.24	2.50	0.00	5.17	9.24	-4.07
40S ribosomal protein S4, X isoform	7.00	5.00	4.00	3.00	11.00	8.00	3.00
Junction plakoglobin	9.00	7.00	4.00	4.00	13.00	11.00	2.00
Heterogeneous nuclear ribonucleoprotein K	2.00	2.00	1.00	0.00	3.00	2.00	1.00
40S ribosomal protein S11	2.00	2.00	1.00	0.00	3.00	2.00	1.00

Propionyl-CoA carboxylase beta chain, mitochondrial	4.00	3.00	1.00	2.00	5.00	5.00	0.00
40S ribosomal protein S3a	3.00	3.00	2.00	2.00	5.00	5.00	0.00
60S ribosomal protein L7a	3.00	4.00	1.00	0.00	4.00	4.00	0.00
Histone H4	4.00	4.00	3.00	3.00	7.00	7.00	0.00
40S ribosomal protein S24	2.00	2.00	0.00	0.00	2.00	2.00	0.00
60S ribosomal protein L19	2.00	2.00	0.00	0.00	2.00	2.00	0.00
Keratin, type I cytoskeletal 17	0.00	0.00	0.00	0.00	0.00	0.00	0.00
Keratin, type I cytoskeletal 15	0.00	0.00	0.00	0.00	0.00	0.00	0.00
Keratin, type II cytoskeletal 73	0.00	0.00	0.00	0.00	0.00	0.00	0.00
Keratin, type II cytoskeletal 75	0.29	0.14	0.00	0.14	0.29	0.29	0.00
Tubulin alpha-8 chain	0.00	0.33	0.00	0.00	0.00	0.33	-0.33
Tubulin alpha-1C chain	0.00	0.33	0.00	0.00	0.00	0.33	-0.33
Dihydropyrimidinase-related protein 3	0.00	1.00	2.67	2.00	2.67	3.00	-0.33
Keratin, type II cytoskeletal 6A	0.43	0.43	0.00	0.45	0.43	0.88	-0.45
Alpha-enolase	0.00	2.00	1.00	0.00	1.00	2.00	-1.00
60S ribosomal protein L6	2.00	3.00	2.00	2.00	4.00	5.00	-1.00
60S ribosomal protein L17	2.00	2.00	2.00	3.00	4.00	5.00	-1.00
40S ribosomal protein S3	1.00	3.00	1.00	0.00	2.00	3.00	-1.00
Keratin, type I cytoskeletal 12	0.00	1.04	0.00	0.00	0.00	1.04	-1.04
Keratin, type I cytoskeletal 19	0.00	1.40	0.33	0.00	0.33	1.40	-1.07
60S ribosomal protein L7	2.00	3.00	2.00	3.00	4.00	6.00	-2.00
60S ribosomal protein L35	1.00	3.00	2.00	2.00	3.00	5.00	-2.00
Peroxiredoxin-2	1.00	2.00	0.00	1.00	1.00	3.00	-2.00
Guanine nucleotide-binding protein G(o) subunit alpha	0.00	2.00	1.00	1.00	1.00	3.00	-2.00
40S ribosomal protein S20	2.00	2.00	0.00	2.00	2.00	4.00	-2.00
40S ribosomal protein S23	1.00	2.00	0.00	1.00	1.00	3.00	-2.00
60S ribosomal protein L10a	1.00	2.00	0.00	1.00	1.00	3.00	-2.00
60S ribosomal protein L38	1.00	3.00	1.00	1.00	2.00	4.00	-2.00
ADP/ATP translocase 1	0.00	3.00	1.00	0.00	1.00	3.00	-2.00
Neuronal migration protein doublecortin	0.00	2.00	0.00	0.00	0.00	2.00	-2.00
Ras-related protein Rab-6A	1.00	2.00	0.00	1.00	1.00	3.00	-2.00
Keratin, type II cytoskeletal 1b	2.04	3.13	0.00	1.05	2.04	4.17	-2.13
Hemoglobin subunit beta-1	0.00	1.00	0.00	2.00	0.00	3.00	-3.00
Glyceraldehyde-3-phosphate dehydrogenase	1.00	2.00	2.00	4.00	3.00	6.00	-3.00
Histone H1.4	0.00	2.00	0.00	1.00	0.00	3.00	-3.00
60S ribosomal protein L9	1.00	1.00	0.00	3.00	1.00	4.00	-3.00
ATP synthase subunit gamma, mitochondrial	1.00	1.00	0.00	3.00	1.00	4.00	-3.00
Triosephosphate isomerase	1.00	2.00	2.00	4.00	3.00	6.00	-3.00
60S ribosomal protein L4	3.00	7.00	3.00	2.00	6.00	9.00	-3.00

40S ribosomal protein S8	1.00	4.00	2.00	2.00	3.00	6.00	-3.00
40S ribosomal protein S25	0.00	2.00	0.00	1.00	0.00	3.00	-3.00
ATP synthase subunit alpha, mitochondrial	4.00	5.00	4.00	7.00	8.00	12.00	-4.00
40S ribosomal protein S13	0.00	3.00	0.00	1.00	0.00	4.00	-4.00
40S ribosomal protein S6	1.00	2.00	0.00	3.00	1.00	5.00	-4.00
F-box only protein 6	1.00	3.00	1.00	4.00	2.00	7.00	-5.00
Tubulin beta-2B chain	5.33	4.33	2.50	8.60	7.83	12.93	-5.10
Actin, cytoplasmic 1	7.00	10.00	4.80	7.00	11.80	17.00	-5.20
Tubulin beta-3 chain	2.00	5.43	2.00	4.40	4.00	9.83	-5.83
ATP synthase subunit beta, mitochondrial	4.00	7.00	2.00	5.00	6.00	12.00	-6.00
Tubulin alpha-1A chain	5.00	13.00	6.67	5.00	11.67	18.00	-6.33
Methylcrotonoyl-CoA carboxylase subunit alpha, mitochondrial	17.00	20.00	11.00	20.00	28.00	40.00	-12.00
Propionyl-CoA carboxylase alpha chain, mitochondrial	11.00	22.00	13.00	16.00	24.00	38.00	-14.00
Pyruvate carboxylase, mitochondrial	26.00	50.00	22.00	32.00	48.00	82.00	-34.00
Acetyl-CoA carboxylase 1	58.00	80.00	42.00	76.00	100.00	156.00	-56.00

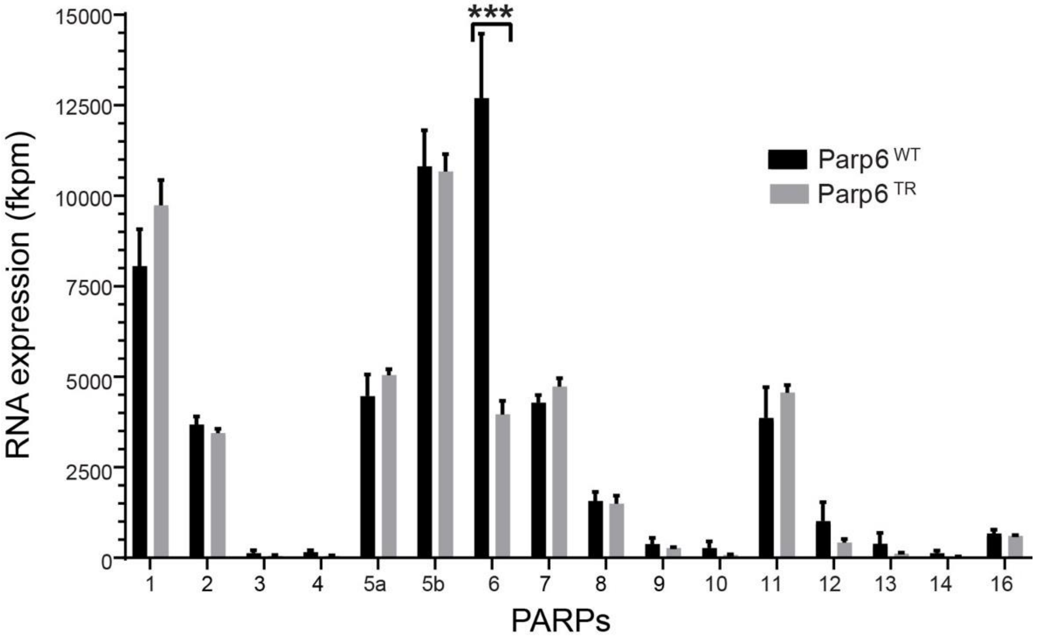

Figure S1: **CRISPR-Cas9 mutagenesis resulted in *Parp6* transcript knock down, while the other PARPs were not affected.** Briefly, total RNA obtained from Parp6^{WT} and Parp6^{TR} E18 embryonic hippocampi (n=4 each) was purified and sent to the Massively Parallel Sequencing Shared Resource (OHSU) for RNAseq, where it was quality-assessed, sequenced and analyzed. Graph shows the average count (fkpm) of the samples for the different *PARP* transcripts (mean ± S.E.M.). Significance was calculated by T-tests (*** = p<0.0001).

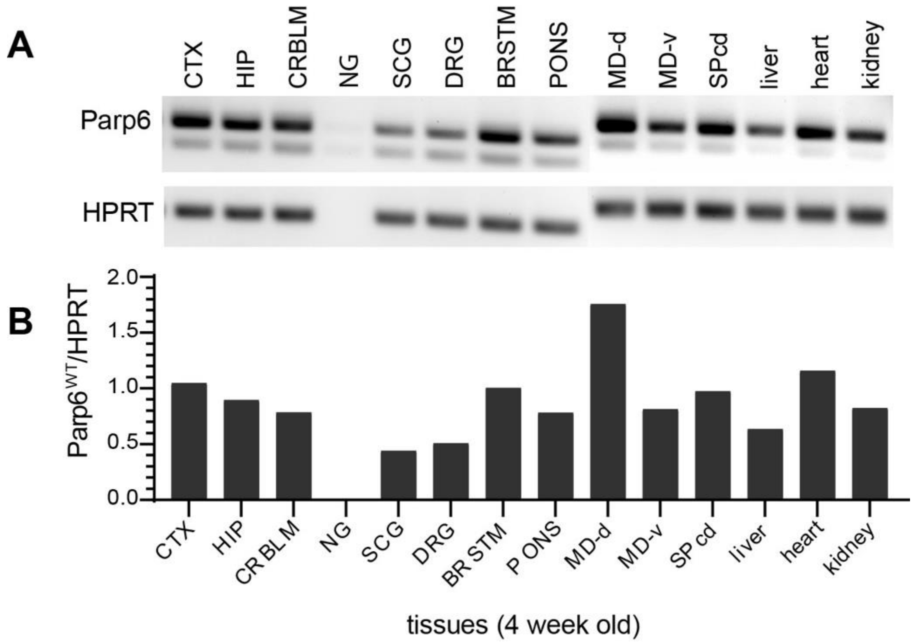

Figure S2: ***Parp6^{WT}* transcripts were found to be highest in the dorsal medulla of the brainstem in 4-week-old mice.** Transcript expression was assessed by RT-PCR (see general method section 2.5 for details). *HPRT* transcripts were used as internal control. Abbreviations: CTX: cortex, HIP: hippocampus, CRBLM: cerebellum, NG: nodose ganglion, SCG: superior cervical ganglion, DRG: dorsal root ganglion, BRSTM: brainstem, MD-d: dorsal medulla, MD-v: ventral medulla, SPcd: spinal cord

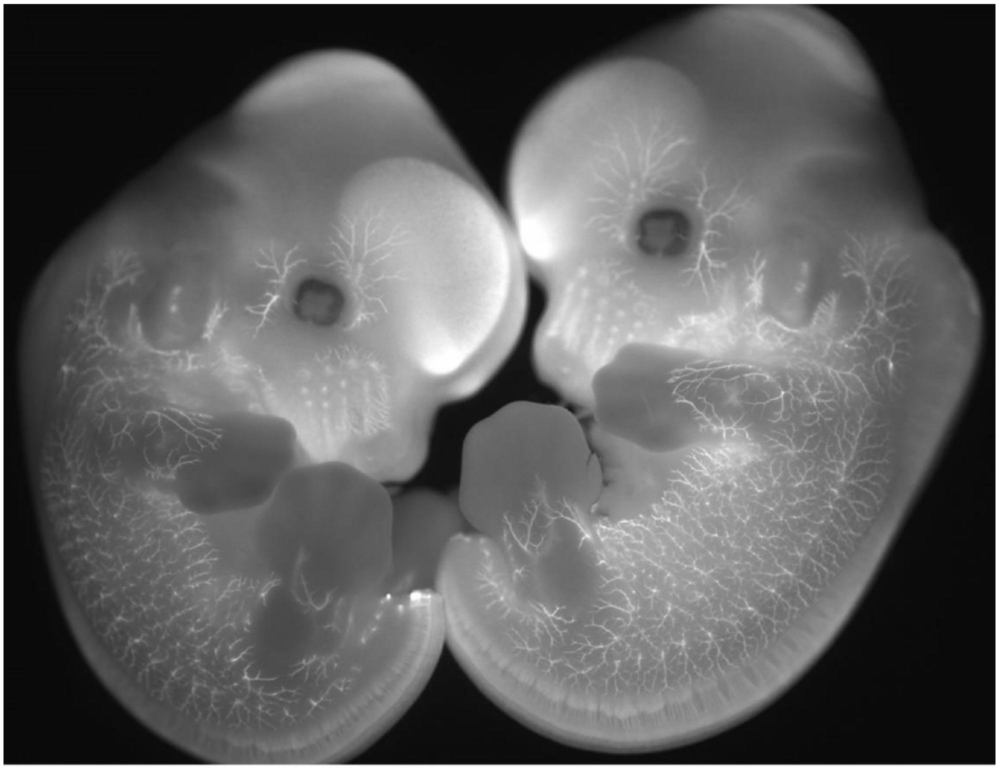

Figure S3: Immuno-labeled and cleared Parp6^{HT} and Parp6^{TR} E12.5 embryos were imaged and overall embryo development was quantified through eye and paw area measurements. Peripheral nerved development was quantified through outgrowth measurements of the forepaw axon bundle and intercostal axon bundle outgrowth (see methods and Table **S5** for measurements).

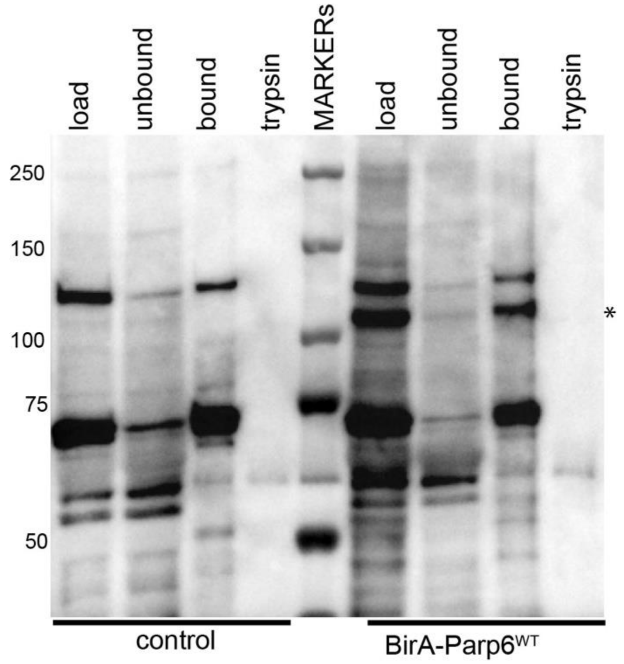

Figure S4: Biotinylation of proximal Parp6 interactors by Myc-BirA*–Parp6 in the presence of biotin. Cortical neurons cells were transduced with Myc-BirA*–Parp6 and treated with media supplemented with or without biotin for 24 h. Biotinylated proteins were visualized using streptavidin−HRP (Biotin). (*) indicates BirA-Parp6 protein.

Video S1: **Video of the locomotion of two Parp6^{TR} mice compared to four Parp6^{WT} and Parp6-^{HT} siblings.** All mice are post-natal day 23 (P23) siblings. Smaller mice are Parp6^{TR}, note their twitchy and hesitating movements.
